# CRIMALDDI: platform technologies and novel anti-malarial drug targets

**DOI:** 10.1186/1475-2875-12-396

**Published:** 2013-11-05

**Authors:** Henri Vial, Donatella Taramelli, Ian C Boulton, Steve A Ward, Christian Doerig, Kelly Chibale

**Affiliations:** 1 Centre National de la Recherche Scientifique, UMR 5235, Université Montpellier 2, Place Eugene Bataillon, 34095 Montpellier, Cedex 5, France; 2 Dipartmento di Scienze Farmacologiche e Biomolecolari, Università di Milano, Via Pascal 36, 20133 Milano, Italy; 3 TropMed Pharma Consulting Ltd, 10 Brampton Chase, Lower Shiplake, Oxfordshire RG9 3BX, UK; 4 Liverpool School of Tropical Medicine, Pembroke Place, Liverpool L3 5QA, UK; 5 Department of Microbiology, School of Biomedical Sciences, Monash University, Wellington Road, Clayton VIC 3800, Australia; 6 Department of Chemistry, University of Cape Town, Rondebosch, Cape Town 7701, South Africa

**Keywords:** CRIMALDDI, Malaria, Drug discovery, Research agenda, Novel targets, Enabling technologies, Prioritization

## Abstract

The Coordination, Rationalization, and Integration of antiMALarial drug Discovery & Development Initiatives (CRIMALDDI) Consortium, funded by the EU Framework Seven Programme, has attempted, through a series of interactive and facilitated workshops, to develop priorities for research to expedite the discovery of new anti-malarials. This paper outlines the recommendations for the development of enabling technologies and the identification of novel targets.

Screening systems must be robust, validated, reproducible, and represent human malaria. They also need to be cost-effective. While such systems exist to screen for activity against blood stage *Plasmodium falciparum*, they are lacking for other *Plasmodium* spp. and other stages of the parasite’s life cycle. Priority needs to be given to developing high-throughput screens that can identify activity against the liver and sexual stages. This in turn requires other enabling technologies to be developed to allow the study of these stages and to allow for the culture of liver cells and the parasite at all stages of its life cycle.

As these enabling technologies become available, they will allow novel drug targets to be studied. Currently anti-malarials are mostly targeting the asexual blood stage of the parasite’s life cycle. There are many other attractive targets that need to be investigated. The liver stages and the sexual stages will become more important as malaria control moves towards malaria elimination. Sexual development is a process offering multiple targets, even though the mechanisms of differentiation are still not fully understood. However, designing a drug whose effect is not curative but would be used in asymptomatic patients is difficult given current safety thresholds. Compounds active against the liver schizont would have a prophylactic effect and *Plasmodium vivax* elimination requires effectors against the dormant liver hypnozoites. It may be that drugs to be used in elimination campaigns will also need to have utility in the control phase. Compounds with activity against blood stages need to be screened for activity against other stages.

Natural products should also be a valuable source of new compounds. They often occupy non-Lipinski chemical space and so may reveal valuable new chemotypes.

## Background

There has been significant progress in recent years towards the global goal of malaria elimination. However, the recent reports of artemisinin resistance in Southeast Asia have emphasized the continuing need for a wide range of anti-malarials with novel chemical scaffolds and modes of action. This is needed to adequately address the challenges to current drugs and extend the armoury to those aspects that are not well served by current drugs (especially against *Plasmodium vivax* malaria and transmission of the disease). The ultimate goal of elimination demands a range of new drugs must be developed quickly to meet very different challenges compared to control.

A major challenge for successful anti-malarial drug discovery is to quickly identify druggable targets and leads. This requires improved methods to study the various stages of the parasite life cycle (e, g*.* liver and blood stages, sexual forms, and midgut sporozoites) so as to find possible new drug targets. Some species and/or life cycle stages are difficult to maintain or produce *ex vivo*. Research on human malaria is hampered by technological difficulties that limit the study of parasite biology on all malaria stages and disease pathogenesis. Specialized laboratories or infrastructure ‘platforms’ are thus required to provide access to particular life cycle stages, and these methods need to be robust enough to be used in a wide range of laboratories.

As the objective of malaria treatment moves from control to elimination [1,2], the types of drugs required are changing. Drugs that block the infectivity of the mature sexual form of the gametocyte [3] and or the dormant hepatic form (hypnozoites) of *P. vivax*[4] will be particularly important. There will be questions about the balance between further exploiting existing drug targets and investing in identifying potential new ones, given the high costs/risks associated with new drug discovery and development. Other unanswered questions include the best way to identify new drug targets, whether targets that are common to several life-cycle stages are better than stage-specific targets, and how best to evaluate and prioritize new candidate compounds.

Most currently available anti-malarials target the asexual blood stages of the parasite’s life cycle, the only stages for which there are robust, reproducible, and high-throughput *in vitro* screening methods. However, control and elimination will require new classes of anti-malarial drugs active against all stages of all five species of human malaria. Especially for elimination, new drugs are needed to eliminate all malaria parasites, including the *P. vivax* hypnozoites to prevent relapse, and to interfere with malaria transmission by eliminating gametocytes. Techniques to rapidly screen large libraries of compounds against targets at all the stages of the parasite life cycle of all relevant *Plasmodium* species are not currently available.

As described elsewhere, the Coordination, Rationalization, and Integration of antiMALarial drug Discovery & Development Initiatives (CRIMALDDI) Consortium was established to develop a prioritized set of recommendations to expedite anti-malarial drug discovery [5]. This paper summarizes the findings of two of the Consortium’s workshops organized to develop a set of consensus recommendations regarding enabling technologies and identifying novel targets. Full reports on the workshops can be found on the CRIMALDDI website [6] and in the related paper accompanying this one [7].

## Developing enabling technologies

### General requirements for screening methods

**Figure 1:**
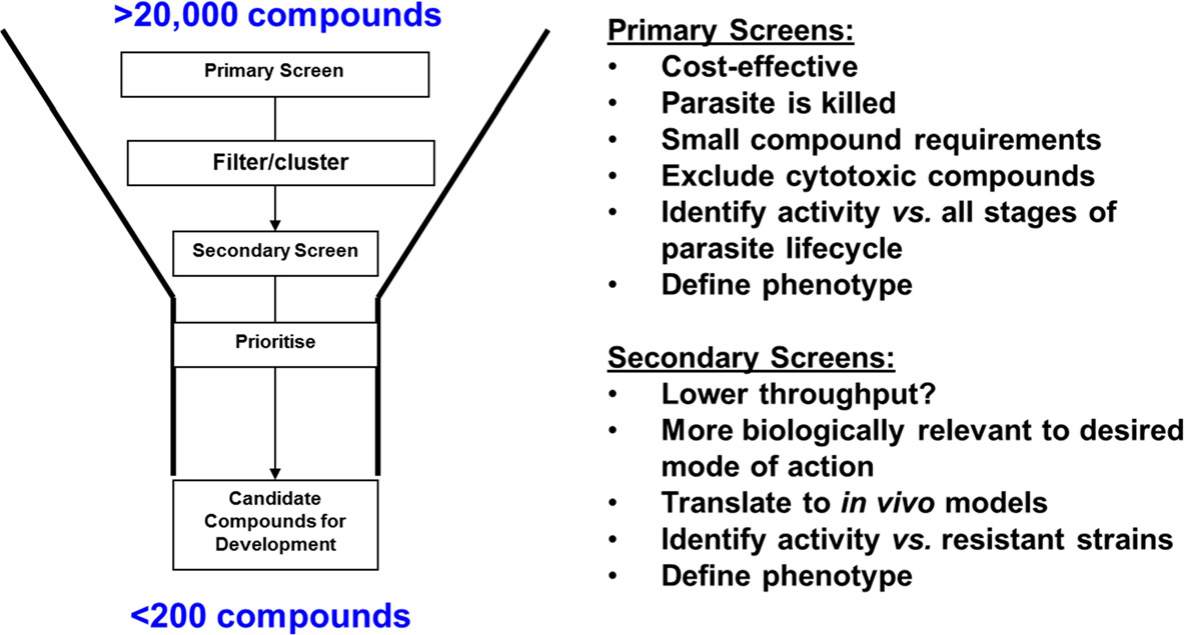
**Two-stage screening process for activity against the various stages of the *Plasmodium *** **parasite life cycle.**

Screening to identify potential drug development compounds active against the various stages of the *Plasmodium* parasite life cycle can be viewed as a two-stage process (Figure 1). Primary screens must be simple enough to be used easily in high throughput screening systems (HTS), whereas secondary screens can be more complex and have only medium throughput capacity. For blood stage *Plasmodium falciparum* positive HTS hits, the current need is to filter >20,000 compounds from HTS whose structures are in the public domain down to <200 compounds to be considered for further development [8].

Primary screening methods must be robust, validated, reproducible, and represent human malaria (Table 1). Whole cell assays are a good way of identifying chemotypes with cellular permeability properties compatible with accessing their targets inside the parasite in its various stages. Screening systems must accommodate inherent strategies to minimise false negative results.

**Table 1:** Essential characteristics of screening assays for novel compounds effective against the different stages of malaria parasite life cycle

Robust	Ensures that screens are easy to perform, low cost, suitable for screening a large variety of chemotypes or natural products.
Validated	Ensures that the results reflect well the activity of the compound against the targeted parasite stage.
Reproducible	Ensures that the results obtained from different laboratories are directly comparable.
Representative of human malaria	Ensures that the results actually reflect the probable action of the compound(s) when used to treat human malaria.

Methods primarily designed to determine the mode of action of a chemotype can be more complex and are implemented in later stages of the drug development process.

Additional requirements of primary screening should include:

• Cost-effectiveness;

• Biologically appropriate, i.e. the compounds actually kill the parasite (cidal rather than cytostatic);

• Small amount of compound needed for the screen;

• Very early exclusion of compounds cytotoxic against the human host;

• The screening process must include reliable models for all stages of the parasite’s life cycle;

• Candidate compounds can either be schizonticidal plus active against another stage, or only active against another stage. Purely schizonticidal drugs are probably going to be of less interest as malaria control and elimination progresses. Primary screening must take this into account. However the rapid spread of resistance to current first-line drugs will require the deployment of curative agents that will probably be schizonticidal.

Secondary screening of positive hits from a primary screens can be more biologically informed and related to the desired activity of the target profile being pursued. Physiochemical and drug-like properties (including toxicological profile and pharmacokinetic parameters) must be considered since they should allow translation to *in vivo* models. Secondary screening must also look for activity against drug-resistant strains of *Plasmodium* and the capacity to withstand resistance. At present, routine screening is performed against parasite strains that obviously do not represent the strains circulating in the population. Screens need to have early access to isolates from patients, especially those showing resistance to important agents (such as artemisinin derivatives), and the culture-adapted *P. falciparum* and *P. vivax* isolates used need to be updated regularly and promptly to reflect the changing parasite population in the field.

All screening methods need to be validated and reproducible between centres to ensure comparability of results [9].

### Screening methods against blood stage parasites

The rapid identification and progression to drug development of compounds active against acute blood stage *P. falciparum* infections was made possible by the availability of an adequate range of *in vitro* screening assays and animal models. In contrast, there are significant gaps in the platform technologies necessary for investigating other species of *Plasmodium*, particularly *P. viv*ax (Table 2).

**Table 2:** **Existing range of *in vitro *** **screening methods and *in vivo *** **models for blood stage anti-malarials**

	***In vitro *** **pharmacology (HTS Screens)**	***In vivo *** **pharmacology**	**Human pharmacology**
*Plasmodium falciparum*	• Radioactive (3H-Hypoxanthine)	Human parasite models:	Human challenge
		• *P. falciparum* in SCID mouse	
	• Colorimetric (pLDH, HRPII)	• *Aotus*/*Saimiri* NHP	
		Mouse models:	
	• Fluorescent (Sybr Green)	• *Plasmodium berghei* (including GFP or Luc reporter strains)	
	• Transgenic parasite (GFP, Luc reporter strains)		
		• *Plasmodium vinckei*	
		• *Plasmodium chabaudi*	
		• *Plasmodium yoelii*	
*Plasmodium vivax*	Invasion assay	Human parasite models	
	Flow cytometry (both at present only medium throughput)	• *Aotus*/ *Saimiri* NHP	
		Primate models	
		• *Plasmodium cynomolgi*	

#### Plasmodium falciparum

Along with the classic radioactive HTS screen for *P. falciparum* using ^3^H-hypoxanthine, colorimetric (pLDH, HRPII) [10-13] or fluorescent (SybrGreen) [14] assays have been widely used. Recently screens using transgenic parasites (GFP, Luciferase reporter strains) have been adapted to HTS [15-17]. At present, there is little need to prioritize additional screening methods for *P. falciparum*. However, there is the requirement to continually upgrade the existing assays. With the emergence of artemisinin resistance [18], compounds that, like artemisinins, are active against the early ring stages of the erythrocytic cycle or those that are active against artemisinin-resistant strains of *P. falciparum* should be a priority. A low throughput viability-based assay approach that allows discrimination between compounds based on their anti-malarial mode-of-action has been developed [19]. Further work is needed to develop more novel screens or further modifications of existing methods.

*In vivo* models using rodent *Plasmodium* species or *P. falciparum* in severe combined immunodeficiency **(**SCID) mice or non-human primates (NHP) are available for pharmacological studies. Mouse models may give species dependent screening results and so different *Plasmodium* species need to be used in the initial rodent model screening of compounds. Primate models are difficult, of limited availability, and not cost-effective.

There is a need to identify a good *in vivo* model for severe malaria that can be used in secondary screening. To find appropriate models, the target profile needs to be better defined.

#### Plasmodium vivax

Discovery of new drugs active against *P. vivax* blood stage infections is being impaired because of a lack of HTS assays (Table 2). The development of such drugs will be key to achieving malaria control and elimination.

The key challenge is the lack of an *in vitro* culture system for growing *P. vivax*[20]. The availability of fresh isolates relies on the collection of infected human blood, which needs to be processed within six hours from collection. Consequently, the laboratory facilities must be beside the patients and in easily accessible locations. In addition, *P vivax* preferentially invades reticulocytes which are short-lived and represent only approximately 1% of the circulating red blood cells. New improved *ex vivo* methods for isolation and cryopreservation of *P. vivax* isolates and of concentrated cord blood reticulocytes have been developed [21] and a short term (24 hour) invasion assay has been standardised and used for both drug sensitivity tests and proteomics [22]. However, the invasion assay still relies on microscopic determination of schizont maturation and reticulocytes invasion and thus is quite laborious and requires trained personnel. More recently, the same group [23] developed a simple, field optimised protocol using fluorescent dyes and flow cytometry to determine the antimalarial drug sensitivity in freshly isolated *P. vivax:* this became possible mostly because portable, low cost cytometers became available. Flow cytometry may become a viable and practical alternative to microscopy, although the problem of long term cultures of *P. vivax* persists and represents a key block to future progress in a number of areas of *P. vivax* research. The development of a SCID mouse model for *P. vivax* could be seen as an alternative.

Virtually all *P. falciparum* blood stage drugs are also active against *P. vivax*, but the levels of sensitivity of each species to the drugs may differ. The workshop concluded that developing primary screens that would pick up vivax activity separately was not a priority. It should be to develop good secondary *in vivo* models, particularly for compounds that have a differential *in vitro* activity against both species.

### Screening methods against liver stage parasites

Until recently, validated screening assays for liver stage *P. falciparum* and *P. vivax* liver stages were virtually non-existent, and much work needs to be done to develop appropriate validated methods (Table 3).

**Table 3:** **Existing *in vitro *** **and *in vivo *** **models against liver stage parasites**

	***In vitro***	***In vivo***	**Human**
	**pharmacology**	**pharmacology**	**pharmacology**
	**(HTS Screens)**		
*Plasmodium* that do not form hypnozoites	• *P. falciparum*	Mouse models	Human challenge
	• *P. berghei*	• *P. berghei*	
	• *P. yoelii*	• *P. yoelii*	
Hypnozoites –forming *Plasmodium* species	• *P. vivax* in hepatocyte cell lines	Primate models	
		• *P. cynomolgi*	
	• *P. cynomolgi* in hepatocytes from Rhesus monkeys		

A major priority for the liver stages of both *P. falciparum* and *P. vivax* is to develop a reproducible and robust hepatocyte culture system that is highly susceptible to large scale invasion by malaria sporozoites. This should ensure the complete development of the parasite and ideally give good machinery for drug metabolism. Another block is the lack of a robust supply of sporozoites and cryogenic methods to store and transport them.

#### Plasmodium falciparum

Rudimentary assays already exist for liver stages that do not form hypnozoites: *P. falciparum*, *Plasmodium yoelii,* and *Plasmodium berghei*[24]. Until recently, only primary hepatocytes appeared suitable for *P. falciparum* and they have very low permissivity for infection [25]. Stable and infectable hepatocyte lines and standardized conditions for culturing hepatocytes need to be developed, because primary human liver cells are of variable quality and lose their differentiation in culture. There is also a priority to find ways of increasing the permissivity of hepatocytes to infection by sporozoites. The recent study by Bathia and colleagues that use cryopreserved hepatocytes (described below for *P. vivax* but also applied to *P. falciparum)* is of potential value for all liver stage models [26].

#### Plasmodium vivax

The situation with screening for active liver stage activity against *P. vivax* infections is the same as for *P. falciparum,* with the additional challenges of (a) developing methods to cultivate the sexual blood stage (and thus to obtain infective sporozoites following mosquito feed) and (b) developing appropriates tools to distinguish the hypnozoite from the replicating schizont stage. Clearing hypnozoites is critical for malaria elimination.

Cellular assays for the three key steps of liver-stage biology are thus needed: hepatocyte infection, hypnozoite formation, and reactivation to hepatic schizonts. Hypnozoite identification is difficult because of their relatively small size, absence of validated biomarkers and the low infection rate [4,24]. A small-scale liver culture system for *P. vivax*, relying on cryopreserved sporozoites has identified primaquine-sensitive small forms [27]. Recently, schizonts and fluorescent uninucleate persisting forms (displaying all the characteristics of hypnozoite-forms) could be characterized for *Plasmodium cynomolgi*, a simian parasite related to *P. vivax*. These were observed after sporozoite infection of *Macaca fascicularis* primary hepatocytes [25,28]. This simian system provides a more reliable supply of both parasites and primary cells as well as transgenic fluorescent parasites. Another recent study has reported an *in vitro* model that recapitulates the entire liver stage for both P*. falciparum* and *P. vivax* in a microscale human liver platform composed of cryopreserved, micropatterned human primary hepatocytes surrounded by supportive stromal cells. This system allow the release of infected merozoites and infection of overlaid erythrocytes, as well as the establishment of small forms in late liver stages of *P. vivax*[26].

Biomarkers that distinguish hypnozoites from the hepatic schizonts are needed to speed up work on the nature of dormancy. There is very little understanding of the biology of both the liver schizont and the hypnozoite stage of infection. The pressing question is “what is a hypnozoite, how does it function, and how can its quiescent metabolism be targeted?”

The availability of robust and reliable culture models and methods for hypnozoite identification will provide the foundations to obtain key information on the biology of hypnozoite and their reactivation process, paving the way for a quantitative high-throughput liver stage screen [29]. Once appropriate screens have been developed, compounds will be tested first for activity against all hepatic stage in a primary screen and then secondarily screened for hypnozoite activity.

### Screening methods against sexual stages

Transmission blocking (through the sexual and mosquito-based stages of the *Plasmodium* life cycle) will become more important as malaria control moves towards malaria elimination. It is argued that the number of parasites that need to be killed to break the transmission cycle is orders of magnitude less than is the case in treating infections.

Currently there is no generally accepted detailed target profile for a new chemical entity that would act principally against the sexual stages. Such a profile needs to be developed so that it is clear exactly which stage(s) should be targeted (gametocytes, intermediate stages – gametes, zygotes, ookinetes, oocysts, or finally sporozoites). Recently, a comprehensive review on the next generation of antimalarial drugs needed to control and eliminate malaria has been published and the target candidate profiles (TCP) for transmission blocking agents have been outlined [30].

Most probably conventional blood schizonticides will block the transition from asexual to sexual stages, so screening for drugs that block this transition is not the first priority. However, mature gametocytes are long-lived and even patients on treatment may continue to transmit malaria for weeks after clearance of asexual parasites. There is data showing that some anti-malarial drugs can in fact induce gametocytogenesis [31]. Drugs that will kill long-lived stage V gametocytes and/or extra-erythrocytic parasite stages are needed to sustain the elimination phase. Over the last few years, efforts have been made to develop a reproducible and robust method for producing gametocytes *in vitro,* and sustain their *in vitro* differentiation until the long-lasting late stage V. In addition, new assays for gametocytocidal activity have been developed and are presently used for large compounds screening (Table 4) [32]. These assays fall into three major categories:

1. Measurements of parasite DNA content using fluorescent dyes (like hydroethidine) for flow cytometry [34]. The sensitivity is good but the throughput is quite low. High content screening assays for stage IV-V gametocytes are under development [42].

2. Measurement of the metabolic/redox activity of *P. falciparum* gametocytes, using the ATP bioluminescence assay for late stage gametocytes [36,37], the fluorimetric Alamar blue test [39,40], or the colorimetric assay with pLDH [41]. The advantage of all these assays is that they can be used with common laboratory strains or even field isolates since they do not require transgenesis.

3. Transgenesis has been applied with success to produce *P. falciparum* lines expressing GFP or GFP-Luc reporter genes active on different stages of gametocyte maturation. They have been used to identify compounds active on early (I-II), middle (III) or late (IV-V) stage gametocytes [35]. The GFP transgenic lines have been used with flow cytometry [37,38]. The assays using GFP or GFP-Luc reporter genes appear to be quite sensitive, robust, with a good signal to noise ratio.

**Table 4:** **Existing *in vitro *** **assays for screening compounds against *Plasmodium falciparum *** **gametocytes (stage I-V)**

***In vitro *** **pharmacology**	***In vitro *** **pharmacology**
**L/MTS/HTS Screens (using non-transgenic parasites)**	**MTS/HTS screens (using transgenic parasites)**
Microscopic counts of Giemsa smear (gold standard)	Pfs16-GFP transgenic parasites [33].
Flow cytometry of hydroethidine labelled gametocytes [34]	Stage-specific luciferase expression in transgenic parasites [35]
ATP- bioluminescence assay for stage IV-V gametocytes [36,37]	Three transgenic GFP lines, used for flow cytometry assay [38]
Alamar blue, fluorescent redox indicator [39,40]	
Parasite lactate dehydrogenase activity (pLDH) [41]	

### Assays against the sporogonic stages in the mosquito

To achieve the objective of local elimination/eradication of malaria, it is well recognized that malaria transmission through the mosquito vector must be reduced. The standard membrane-feeding assay (SMFA) is the gold standard assay for evaluation of any transmission-blocking intervention, but it has a very low throughput and is expensive.

A series of novel assays (Table 5) that can identify activity across almost all sexual stages have been recently described or are under development. Some of them are still in a medium or low throughput format, but improvements are expected shortly. The *P. berghei* ookinete development assay is the only one adapted to HTS format [43]. The test is quite robust, and reproducible and has been used in HTS screens of a large compound library (the Tres Cantos Antimalarial Set (TCAMS)) [44].

**Table 5:** Existing assays for screening transmission blocking compounds on the sporogonic stages in mosquitoes

**Pharmacology**	***Plasmodium *** **species**
**L/MTS/HTS Screens**	
Male exflagellation assay [45]	*P. falciparum*
Female activation assay (Delves, unpublished)	*P. falciparum*
Standard membrane feeding assay (SMFA) [46,47]	*P. falciparum*
SMFA using PbGFP CON [48]	*P. berghei*
	*Anopheles stephensi*
*P. berghei* ookinete development assay [43]	*P. berghei* (transgenic)
GFP-luc sporozoites in mosquito salivary glands [49]	*P. berghei* (transgenic)

To verify if stage V gametocytes are able to continue their differentiation toward male and female gametes, two different assays are available. The *P. falciparum* exflagellation assay is limited to male gametocytes, and although it has recently been semi-automatized by image acquisition of motile ‘exflagellation’ events, still it is limited by the short time-window of this phenomenon [43].

The production of female gametes can be visualized by morphology on Giemsa-stained smears. The stable expression of unique surface antigens by female gametes has offered the possibility to develop MTS/HTS assays of macrogametogenesis (Delves *et al.* unpublished).

Finally, the availability of GFP-tagged lines is transforming the throughput of the SMFA assay [46], one of the most sensitive, albeit very low throughput, assay to evaluate transmission-blocking compounds [48]. Counting GFP-Luc expressing salivary gland sporozoites in mosquito lysate is also possible, although with limited sensitivity at present and only in the *P. berghei* model [49].

Drugs targeted solely at the late sexual stages will need to reside in the human body for at least five to nine days after the appearance of symptoms. This is in order to be present when the mature gametocytes (Stage V) start to appear in the blood. This has significant implications for the bioavailability and pharmacokinetic properties of drug candidates (see “Priorities for the development of enabling technologies”).

### Priorities for the development of enabling technologies

#### Blood stages

• Understanding the technical problems behind the development of a *P. vivax* blood stage culture system and overcoming them.

• Development of a *P. vivax* blood stage rodent model.

#### Liver stages

• Development of a robust hepatocyte cell system to enable study of *P. falciparum & P. vivax* liver stages.

• Development of a standardised and improved culture system for the study of *P. falciparum & P. vivax* liver stages.

• Understanding of the nature and biology of hypnozoites with the objective of identifying a biomarker for drug activity against this stage of *P. vivax* infection.

• Development of a robust and reliable supply of falciparum and vivax sporozoites and a method for safely transporting them between laboratories.

• Investigate the possibility of using a liver schizont assay system as a surrogate for activity against hypnozoites in primary screening for novel anti-vivax drugs.

#### Sexual stages

• Development of a target product profile for a drug to target the sexual stages of *P. falciparum*.

• Development of robust screening methods for stage IV-V gametocytes of *P. falciparum*

• Development of a *P. falciparum* (and *P. vivax*) ookinete assay to complement the current *P berghei* assay.

• Development of male and female gamete activation assays

• Development of MTS/HTS for SMFA assay

## Identifying novel targets

There is a clear need for the identification and development of new classes of anti-malarials, i.e. belonging to novel chemical series and with novel modes of action. The current reality is that much drug discovery is still focused on a limited number of historic targets (primarily the folate and haemoglobin degradation pathways), and primarily on the blood stage of the parasite’s life cycle. There is a wealth of untapped targets in the various developmental stage proteomes that might be targeted.

Databases on genes and metabolic pathways offer opportunities to identify possible new target-based approaches. The cross-correlation of this information can be a very valuable filter (e g, identifying genes that are active in both gametocytes and in the blood stages). Orthology-based screening between *Plasmodium* species may also give valuable insights into promising new targets. The high level of conservation between *P. falciparum* and *P. vivax* genomes indicates that novel targets for vivax might be established from proven *falciparum* targets.

As the effort against malaria moves from the control phase to elimination, parts of the parasite life cycle beyond the blood stages will need to be targeted. Liver stages, gametocytes and sporozoites were highlighted in the Workshop as being potentially important for the elimination phase.

### Targeting erythrocytic schizogony

In the control phase, the focus is on the treatment of symptomatic patients, i.e., treating asexual blood stages.

#### Finding novel drug classes

One approach is the mining of genomic databases to identify novel targets (see below), followed by the screening of high diversity chemical libraries on such targets. Another strategy consists of first using such libraries (including libraries of natural products) in phenotypic screens to constitute the so-called “malaria boxes”, and subsequently identify in secondary screens the molecular targets of compounds with parasiticidal activity. Several million chemical entities have been screened for anti-malarial activity, based on whole cell screens of *P. falciparum*. The screens have included fully synthetic (from both commercially available and pharmaceutical industry proprietary sources) and natural product libraries. More than 20,000 compounds are now available that are known to have selective parasiticidal activity, but whose molecular targets are unknown [50]. Many of these are likely to act through novel pharmacophores on novel molecular targets. A wider range of libraries should be investigated, including compound banks from agrochemical companies. The accompanying paper outlines the CRIMALDDI Consortium’s recommendations to expedite the use of this valuable source of information [7].

#### Finding novel targets

The characteristics of a good target should include:-

“Must have” characteristics:

• Compound should be able to kill the parasite (be “cidal”) in much less than 48 hours through either inhibition of the target or interaction of the compound with the target;

• The target should be validated either chemically or genetically, both *in vitro* and *in vivo*;

• The target should be druggable, i.e. should be amenable to treatment with small molecules at low concentrations;

• There needs to be a reliable functional assay of the target.

“Nice to have” characteristics:

• Lack of orthology of the target in humans;

• Cellular assay of the effect of compounds on the target;

• A crystal structure of the target.

#### Considerations about the various stages in erythrocytic schizogony

A key requirement in most target product profiles is rapid parasite kill. The speed-of-action of the artemisinins is attributed to their activity throughout the erythrocytic stage (in contrast, most other anti-malarials that are active only in the trophozoite stage). Similarly, interfering with the schizont stage and interrupting the release of merozoites are potentially promising but unexploited strategies.

Other processes identified for further biological understanding that might lead to novel targets of most value for drug discovery were:-

• Ring stage particularly focussing on Merozoite invasion: formation of the parasitophorous vacuole and associated transport pathways.

• Trophozoite stage: Metabolic pathway characterisation in the trophozoite (including haemoglobin degradation) and host cell remodelling.

• Schizonts: Nuclear division, merozoite ontogeny, egress mechanisms.

In the absence of robust culture systems it is reasonable at this stage to assume *P. falciparum* will be a suitable model for blood stage *P. vivax* infections in many cases. However, additional work is needed to fully validate this assumption on a hit by hit basis.

### Host cell targets

Recent research has identified an essential role for host erythrocyte enzymes in parasite development [51,52]. A number of the parasiticidal compounds may target a host cell enzyme rather than a molecule encoded by the parasite. Targeting host cell (erythrocyte or hepatocyte) enzymes that are required for parasite survival is an additional, as yet unexploited, therapeutic strategy. Recent research indicates that such potential host cell targets include enzymes that are well-established targets in other pathologies (e g, protein kinases in cancer). A “piggy-back” approach might be implemented that would considerably speed up discovery/development. Additional fundamental research must be performed to validate this approach.

### Targeting liver stages

The highest priority in this area is to understand the biology of liver stage parasites, especially the hypnozoites that act as a reservoir for *P. vivax*. Drug activity against hypnozoites will become crucial during elimination. At present even the exact definition of a hypnozoite is not clear. Without such understanding, practical screening techniques cannot be developed and modes of action will be poorly understood. A high priority is to develop simple markers to identify hypnozoites from active, infected hepatocytes (preferably in human liver cells). This would be a key step to developing screens against the dormant stages.

Priority should also be given to the following aspects of the infected hepatocytes:

• Reactivation of hypnozoites in an *in vitro* model to allow testing of compounds which block reactivation or kill the dormant cells;

• Causes of dormancy and the reasons for liver stage parasites to “choose” between the active and dormant state.

Some insights or leads that may be starting points for research were identified:-

• Known efficacy of 8-aminoquinolines (primaquine and tafenoquine) against hypnozoites;

• The use of bio-informatics to identify potentially important genes and metabolic pathways;

• Non-haem targets in the blood stage;

• Effect of antibiotic pre-treatment of apicoplasts.

### Targeting sexual development and sporogony

Gametocytes are an interesting point to interrupt transmission of the parasite. This could be either the sole activity or an additional activity from a compound that is acting elsewhere in the life cycle. There will be some ethical and practical issues: a purely anti-gametocyte drug would not directly benefit the patient and so would impose a high safety requirement on the compound. On the other hand, a drug targeting gametocytes exclusively (but not affecting asexual stages) would be a valuable asset in preventing the escape of genotypes that confer resistance against anti-asexual drugs: thus one of the main benefits of transmission-blocking drugs would be to protect co-administered curative drugs against resistance development and spread. The mechanisms of action of existing, blood stage, active compounds against gametocytes should be investigated.

Recent research has identified crucial roles for several enzymes in sexual development. Possible target processes to attack gametocytes include:

• Gametocytogenesis: a process with many metabolic processes active and so offering multiple points to disrupt the parasite;

• Egress and subsequent gametogenesis mechanisms (especially proteases, protein kinases, and lipid metabolism);

• The mode of action of primaquine against gametocytes and whether it has a common biochemical target at all stages of the parasite life cycle.

Similarly, drugs that have activity against sporozoites might be of value. Sporozoite-active compounds would act in many ways like a parasite repellent. The target product profile should clarify how this type of activity will be of demonstrable value in the field. It will be useful to screen against sporozoites all the current screening hits to see if there is this additional activity to that against blood and/or liver stages.

### Natural products

The chemical diversity derived from natural products is tremendous. Natural products are an “exquisite” source of new drugs as Nature has screened orders of magnitude of more compounds than has been possible with HTS. Many current anti-malarials originated as natural products. However in recent years no compounds in development have originated directly from a natural product. Many natural products have shown potent antiplasmodial effects but have not been progressed [53]. Recent developments (such as the availability of the whole parasite genome) warrant a re-visit of the natural product route of drug discovery *vis-à-vis* target identification. Natural products generally span a different chemical space than synthetic compounds. In addition, natural products can be bio-available with properties outside conventional Lipinski space and they have evolved to interact with biological molecules. Therefore, screening of natural products in whole cell assays are of potentially great value in identifying new druggable targets that would be rejected if the Lipinski rules were blindly followed [54,55]. They will also be of value in identifying polypharmacological modes of action. The metabolic activity identified with natural products can also be used to define empirical targets without initially establishing a protein target [56]. The protein/target may be determined later in the development process. Naturally occurring “libraries” of natural products should not be ignored, both in seeking novel chemical classes and as tools to identify novel targets. They may have a better chance to produce hits in drug screening because they have evolved to interact with biological targets. Another approach by which natural products may be useful in the identification of druggable targets is activity-based protein profiling [57]. Proteins/enzymes identified in this way can then be investigated further as potential therapeutic targets by both classical mechanism of action studies or reverse pharmacology [58].

The shared ancestry of apicoplexans and algae suggest that new “plant-like” targets exist beyond the apicoplast. This may be of value in identifying leads for less metabolically active stages in the parasite life cycle (e g, gametocytes, hypnozoites). The problem of the regulatory pathway with natural product mixtures was noted. The drive for low-cost drugs also was a problem for natural products, which are often complex molecules and have potentially high costs of manufacture. However, better semi-synthetic approaches (as has is being used for artemisinin) may reduce this problem.

## Conclusion

The ambitious target of malaria elimination will only be achieved with the development of new drugs carefully targeted at the key challenges that exist for both control and elimination. The CRIMALDDI Project aimed to identify the most important approaches that should be adopted, as a guide to the various organizations – policy makers, funders, and researchers – on where to prioritize their efforts.

It is clear from the recommendations of the two Workshops described in this paper that a very pressing need is to develop the new and improved enabling technologies that allow new drugs with the right target profiles to be identified quickly. These novel technologies are often forgotten in the understandable drive towards the development of new medicines. At a time of limited resources, it is important that drug discovery efforts are clearly focused on the areas of greatest need and/or which show the greatest probability of success. Better enabling technologies will assist in this prioritization, as well as helping to identify novel approaches and enabling them to be studied more effectively.

Exploring a more diverse range of potential drug targets is a priority. These may be better able to address the shortcomings of the current range of anti-malarials. A broader approach that explores of the entire parasite’s life cycle is recommended, as this holds the promise both of addressing current challenges and meeting the requirements of drugs for elimination [29].

The work of the CRIMALDDI Project described here and in related papers [5,7] will hopefully provide some guidance to policy-makers, funders, and researchers in prioritizing their malarial drug discovery efforts. It can also draw attention to the importance of the interplay between the existence of new technologies and the malaria community’s ability to move beyond the current portfolio of well-characterized and studied targets. It should add to the global discussion on how best to prioritize scarce resources, especially as the community moves into the post-Millennium Development Goal period and an updated Global Malaria Action Plan is being developed.

## Abbreviations

CRIMALDDI: The coordination, rationalization, and integration of antimalarial drug discovery & development initiatives; EU: European Union; HTS: High-throughput screening; MMV: Medicines for malaria venture; MTS: Medium-throughput screening; NHP: Non-human primate; SCID: Severe combined immunodeficiency; SMFA: Standard membrane feeding assay; TDR: UNICEF/UNDP/World Bank/WHO Special Programme for Tropical Disease Research & Training; TPP: Target product profile.

## Competing interests

The authors declare that they have no competing interests.

## Authors’ contributions

HV, DT, CD, and KC prepared the first draft of paper. ICB subsequently edited it. All authors contributed to the preparation of the paper. All authors have read and approved the final manuscript.
